# Janus kinase inhibitor therapy for the treatment of spondyloenchondrodysplasia with immune dysregulation due to novel *ACP5* variants: a multicenter study

**DOI:** 10.3389/fimmu.2026.1788476

**Published:** 2026-04-01

**Authors:** Huilin Mu, Li Liu, Zongwen Chen, Ran Chen, Xin Liu, Junfeng Wu, Xi Yang, Xuemei Tang, Lina Zhou, Yanjun Jia, Pratap Kumar Patra, Nisar Ahmad Wani, Saniya Gupta, Xiaodong Zhao, Aaqib Zaffar Banday, Yunfei An

**Affiliations:** 1 National Clinical Research Center for Children and Adolescents’ Health and Diseases, Ministry of Education Key Laboratory of Child Development and Disorders, Chongqing Key Laboratory of Child Rare Diseases in Infection and Immunity, China International Science and Technology Cooperation base of Child development and Critical Disorders, Children’s Hospital of Chongqing Medical University, Chongqing, China; 2 Tianjin Children’s Hospital (Tianjin University Children’s Hospital), Tianjin, China; 3 Department of Pediatrics, Chongqing University Three Gorges Hospital, Chongqing, China; 4 Department of Rheumatology and Immunology, Children’s Hospital of Chongqing Medical University, Chongqing, China; 5 Department of Pediatrics, All India Institute of Medical Sciences, Patna, Bihar, India; 6 Department of Pediatric Radiology, Government Medical College, Srinagar, Kashmir, India; 7 Department of Endocrinology, Edison Hospital, Mohali, Punjab, India; 8 Department of Pediatrics, Government Medical College, Srinagar, Kashmir, India

**Keywords:** ACP5, autoinflammatory disorders, inborn errors of immunity, Janus kinase inhibitor, spondyloenchondrodysplasia with immune dysregulation, tofacitinib

## Abstract

**Background:**

Spondyloenchondrodysplasia with immune dysregulation (SPENCD) is a rare autosomal recessive disorder caused by biallelic *ACP5* mutations and is characterized by skeletal dysplasia, neurological involvement, and immune dysregulation. According to the latest International Union of Immunological Societies (IUIS) classification, SPENCD is categorized as an inborn error of immunity within the autoinflammatory disorders spectrum; however, targeted therapeutic strategies for immune manifestations remain limited.

**Methods:**

We conducted a multicenter study including five patients from India and from Chongqing and Tianjin, China. Whole-exome sequencing identified six *ACP5* variants across the five patients, including four novel variants. The diagnosis of SPENCD was established based on the genetic findings in combination with characteristic clinical features and radiological manifestations. Three patients received treatment with Janus kinase (JAK) inhibitors. In one representative treated patient (Patient 1), immunological parameters before and after treatment were evaluated using flow cytometry and quantitative PCR.

**Results:**

Four novel *ACP5* variants were identified in this cohort. All three patients treated with tofacitinib (a JAK inhibitor) showed variable but overall clinical improvement. In one representative patient, immunological changes were observed after treatment, including a reduction in CD21^low^ B cells and changes in T helper cell subsets.

**Conclusion:**

This study expands the spectrum of *ACP5* mutations and provides clinical and immunological observations suggesting JAK inhibitor therapy may be considered for immune dysregulation in patients with SPENCD.

## 1 Introduction

Spondyloenchondrodysplasia (OMIM: 271550) was initially described in 1976 as a skeletal dysplasia with vertebral and metaphyseal changes, diagnosed based on radiological characteristics (1). Subsequently, it was designated as an autosomal recessive immune-skeletal dysplasia caused by mutations in the Acid Phosphatase 5 (*ACP5*) gene (2–4). The term spondyloenchondrodysplasia with immune dysregulation was initially used to describe SPENCD patients with prominent immune manifestations and was also referred to as SPENCDI (OMIM: 607944). However, since 2016, Briggs et al. proposed that SPENCDI and SPENCD represent a phenotypic continuum of the same disorder (5), as features suggestive of immune dysfunction were being noted more frequently (4, 6).

Loss of function of tartrate-resistant acid phosphatase (TRAP), encoded by the *ACP5* gene, is related to hyperphosphorylation of osteopontin, which activates osteoclasts and promotes bone resorption (7). Osteopontin (OPN) is a cytokine present in bone-dissolving osteoclasts as well as in antigen-presenting macrophages and dendritic cells (2, 8). Intracellular osteopontin has been identified as a critical regulator of TLR9–MyD88–IRF7–dependent type I interferon production in plasmacytoid dendritic cells (9). Dysregulated osteopontin signaling may therefore contribute to immune dysregulation and enhanced type I interferon responses observed in ACP5-associated disease (3). As SPENCD is classified among type I interferonopathies (10–13), autoimmune manifestations, including systemic lupus erythematosus (SLE), have been reported and were also observed in our cohort.

We identified five patients with disease-causing *ACP5* variants and analyzed their clinical, radiological, and immune manifestations. All exhibited short stature, typical skeletal dysplasia, and immune dysregulation. Three patients received JAK inhibitor therapy due to features suggestive of type I interferonopathy.

## 2 Methods

### 2.1 Sample collection and processing

The study included three Chinese patients and two Indian patients from five unrelated families with *ACP5* mutations. Fresh whole blood samples were collected from Patient 1 (P1) and healthy control subjects after obtaining written informed consent. Age- and sex-matched pediatric controls were not available for all assays; adult healthy donors were used for experiments requiring fresh blood, and pediatric reference data are provided for selected analyses. The study was conducted in accordance with the Declaration of Helsinki and was approved by the Institutional Review Board of the Children’s Hospital of Chongqing Medical University (Approval No. 2021-138). Written informed consent was obtained from the patients or their legal guardians for participation in the study and for publication of identifiable clinical data and images.

### 2.2 Genetic analysis

Genomic DNA was extracted from peripheral blood samples collected from the patients and their parents. Whole-exome sequencing (WES) of Patient 1 was performed by Beijing Zhiin Orient Company. WES of Patients 3 and 4 was performed by MyGenostics (Beijing, China). Patients 2 and 5 were recruited from India, and *ACP5* variants were identified based on available WES data.

### 2.3 Real-time quantitative PCR

Total RNA was extracted from whole blood with the Blood Total RNA Miniprep Kit (Axygen). cDNA was transcribed by using Superscript II RT (Invitrogen). We analyzed 6 ISGs in the blood (IFIT1, IFI27, IFI44L, ISG15, SIGLEC1, RSAD2) as reported (14). β-actin was used for a normalization control. The amplification was performed with denaturation for 15 min at 94 °C followed by 40 cycles, 94 °C for 15 s, and 60 °C for 1 min. The data were analyzed with the 2−ΔΔCt method and results were expressed as fold induction.

### 2.4 Flow cytometric analysis

Peripheral blood mononuclear cells (PBMCs) were isolated from whole blood samples of P1 and healthy controls using Ficoll-Paque density gradient centrifugation.

For cytokine detection, PBMCs were stimulated with phorbol 12-myristate 13-acetate (PMA, 500 ng/mL) and ionomycin (1 μg/mL) for 4 h at 37 °C. Cells were then fixed using Fixation and Permeabilization Solution (BD Biosciences) for 30 min at 4 °C and permeabilized with Perm/Wash Buffer (BD Biosciences) for 30 min at room temperature. Intracellular staining was performed with antibodies against CD3-PerCP (BioLegend), CD8-BV510 (BioLegend), IL-17A-PE (eBioscience), and interferon (IFN)-γ-APC (BioLegend).

For analysis of phosphorylated STAT1 and total STAT1, PBMCs were surface-stained with CD3-BV421 (BioLegend), CD19-FITC (BD Biosciences), CD14-PE-Cy7 (BD Biosciences), and CD56-APC (BioLegend). Cells were then stimulated with IFN-α (1 μg/mL; PeproTech) or IFN-γ (1 μg/mL; PeproTech) for 15 min, fixed with BD Phosflow Fix Buffer I, permeabilized with BD Phosflow Perm Buffer III (BD Biosciences), and stained intracellularly with phospho-STAT1 (Tyr701)-PE or total STAT1-PE antibodies (BD Biosciences).

To identify regulatory T cells (Treg), PBMCs were stained with CD4-FITC (BioLegend), CD45RA-PECY7 (BD Biosciences), and CD25-BV421 (BioLegend), followed by fixation and permeabilization using Fixation/Permeabilization Concentrate, Fixation/Permeabilization Diluent, and Permeabilization Buffer (eBioscience). Cells were then stained intracellularly with FOXP3-PE (Invitrogen) and CTLA-4-APC (BD Biosciences).

For B-cell subset analysis, whole blood samples were treated with Red Blood Cell Lysis Solution and stained with CD19-PerCP-Cy5.5, CD27-APC-Cy7, IgG-PE, IgA-PE, CD21-PE-Cy7 (BioLegend). B-cell subsets were defined as naïve B cells (CD19^+^CD27^-^IgD^+^), unswitched memory B cells (CD19^+^CD27^+^IgD^+^), and switched memory B cells (MBC; CD19^+^CD27^+^IgD^-^).

To identify circulating T follicular helper (Tfh) cells, whole blood samples were stained with CD3-PerCP, CD4-PE-Cy7, CD45RO-APC (BD Biosciences), CXCR5-BV421, and PD-1-FITC (BioLegend). T follicular regulatory (Tfr) cells were identified by staining with CD3-PerCP, CD4-PE-Cy7, CD45RA-FITC, CXCR5-BV421, CD127-PE, and CD25-APC (BioLegend). For identification of Th1, Th2, and Th17 subsets, whole blood samples were stained with CD3-PerCP, CD4-PE-Cy7, CD45RA-FITC, CXCR5-BV421, CXCR3-APC (BD Biosciences), and CCR6-PE (BioLegend).

Representative flow-cytometry gating strategies are shown in **Supplementary Figure 5**. All samples were acquired on a FACSCanto™ II flow cytometer (BD Biosciences), and data were analyzed using FlowJo software.

### 2.5 Statistical analysis

Statistical analyses were performed using GraphPad Prism. Sample sizes (n) indicate independent individuals/samples and are provided in the figure legends. Given the rarity of SPENCD, immunological data are presented descriptively, and no multiple-comparison adjustment was applied.

## 3 Results

### 3.1 Initial presentation, growth retardation, and skeletal characteristics

The reasons for initial medical consultation varied among the five patients. Patient 1 (P1) and patient 2 (P2) presented with developmental delay and short stature. Additionally, P2 presented with bleeding diathesis due to immune thrombocytopenia. Patient 3 (P3) presented with severe systemic infection, including keratitis, uveitis, suppurative otitis media, urinary tract infection, purulent meningitis, and bronchopneumonia. The course was complicated by fungal infection. Patient 4 (P4) sought medical attention due to petechiae scattered on the skin and palate mucosa. Patient 5 (P5) was admitted at 1.7 years of age with a four-month history of persistent fever, in association with generalized rash, right knee joint swelling, and oral ulcerations. The age at first consultation ranged from 0.75 to 15 years.

**Figure 1:**
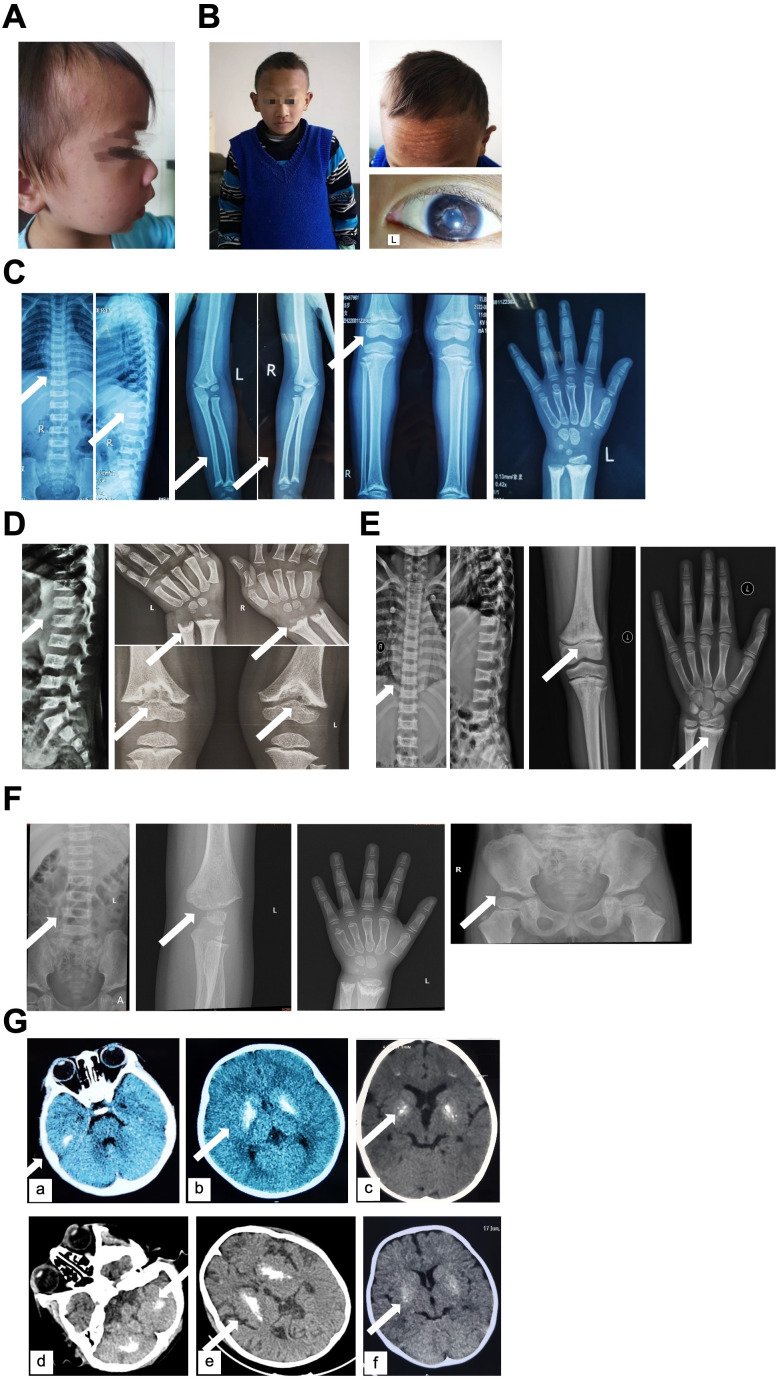
Clinical and radiological features of SPENCD patients. **(A)** Frontal and facial photograph of Patient 1 at the age of 5 years and 6 months. **(B)** Upper-body photograph of Patient 3 at 15 years of age, together with representative images showing frontal facial hyperpigmented lesions and uveitis of the eye. **(C–F)** Representative radiographic and computed tomography (CT) images from Patient 1 **(C)**, Patient 2 **(D)**, Patient 3 **(E)**, and Patient 4 **(F)**. Arrows indicate areas of abnormal bone structure or bone destruction. **(G)** Cranial CT images showing intracranial abnormalities in Patient 1 (a, b), Patient 2 (c), Patient 3 (d, e), and Patient 4 (f).

Two patients exhibited facial abnormalities. P1 had sparse yellow hair, a high hairline, and a broad forehead (**Figure 1A**). P3 presented with a high hairline, thinning hair, a protruding forehead, large ears, dark skin, and uneven pigmentation. He harbored bilateral uveitis, particularly severe in the right eye, resulting in significant visual impairment (**Figure 1B**).

All our patients exhibited varying degrees of growth retardation (**Table 1**). P1 presented significantly delayed height and weight at age 2, along with unexplained postnatal anemia. P2 was 0.75 years old when his developmental delay was first observed. By age 4.5 years, his weight was 10.6 kg and height was 85 cm (both < −3 SD, i.e., more than 3 standard deviations below the age- and sex-matched mean). P3 showed lower height and weight compared to peers since birth. At 15 years old, his height was 127.9 cm (< −3 SD), and weight was 24.9 kg (< −3 SD). Unfortunately, longitudinal height and weight data from birth to age 15 (at hospital visit) were unavailable. P4 had normal growth and motor development during infancy. Weight gain plateaued after her first year, with regression observed between 12 months (9.7 kg) and 18 months (9.5 kg). Her short stature and underweight were first identified during her school age. At 2 years of age, P5 had a height of only 75 cm (< −3 SD) and a weight of 8 kg (< −3 SD), both markedly below the normative range for girls of the same age (**Supplementary Figure 1**).

**Table 1:** Comparison of the clinical manifestations of SPENCD patients.

Items	Patient 1	Patient 2	Patient 3	Patient 4	Patient 5
Age	5.5y	4.5y	15y	9m	2y
Gender	F	M	M	F	F
Disease onset	2 y	0.75 y	–	9 m	1.7 y
Consanguinity	–	+	+	–	–
Similarly affected family member	–	–	–	–	–
*ACP5* variant	c.724C>A, p.H242N	c.del 680-720, p.Leu227Argfs*10	c.791T>C, p.M264T	c.481T>C, p.C161R; c.575T>C, p.L192P	c.550C>T, p.Q184*
Classifications of variants according to ACMG	VUS (variant of uncertain significant)	Pathogenic (PVS1, PM2, PP4)	Uncertain	Uncertain	Pathogenic
IFN-a (in serum)	Increased	N/A	N/A	N/A	N/A
Autoantibody	ANA positive	ANA positive	antiRo-52 antibody positive	ANA positive; dsDNA positive; anti-nucleosome antibodies positive	ANA strongly positive
Weight kg (SD)	14.4 (< −3 SD)	10.6 (< −3 SD)	24.9 (< −3 SD)	8.4	8 (< −3 SD)
Height cm (SD)	97.3 (< −3 SD)	85 (< −3 SD)	127.9 (< −3 SD)	N/A	75 (< −3 SD)
OFC (occipitofrontal circumference) (SD)	N/A	46.3 (<−2 SD)	N/A	N/A	43 (< −3 SD)
Regression	–	–	–	Weight regression (weight 9.7kg at 12m and weight 9.5kg at 18m)	–
Motor delay/milestones	Normal motor and mental developmental milestones with progressive spasticity and gait abnormalities noticed at the age of 2	Global developmental delay (since infancy). Spasticity in all limbs (lower limbs > upper limbs). Inability to walk without support	Delayed motor development (starting to walk with assistance at the age of 2 and walking independently at the age of 2.5). Delayed tooth eruption.	N/A	Delayed
Gait	Abnormal unsteady gait	Cannot walk without support	Normal gait	Normal gait	Normal gait
Cognitive delay	–	Yes	Yes	–	–
1st spoke	12m	Mama, Papa first at 60 months (after 6 months of tofacitinib treatment), before that only monosyllables (monosyllables from 1 year of age)	18m	N/A	9m
Speech	Normal	Delayed speech	Delayed speech	Normal	Normal
Hearing	Normal	Normal	Normal	Normal	Normal
Vision	Normal	Normal	Diminution of vision	Normal	Normal
Facies	Brachycephaly, high anterior hairline, hair thinning and yellowing	–	High anterior hairline, hair thinning, protruding forehead, large ears	–	Normal
Spasticity	–	Spastic paraplegia, hypertonia in upper limbs also. Difficulty in using hands	–	–	Yes (mild)
Deep tendon reflex	Brisk	Brisk	Brisk	N/A	Brisk
EEG	Normal	Normal	Normal	Normal	N/A
Brain CT Scan	Bilateral calcifications in basal ganglia and scattered calcification in bilateral frontal lobe and right parietal lobe	Calcifications in bilateral basal ganglia and subcortical frontal lobes	Calcifications in bilateral frontal lobe, basal ganglia, cerebellar dentate nucleus and pontine area. The ventricular system is slightly widened.	Multiple calcifications in the bilateral basal ganglia and right parietal lobe. The ventricles and extracerebral spaces are widened.	N/A
Brain MRI	N/A	N/A	Bilateral cerebellar dentate nucleus, basal ganglia and lateral ventricle show short T1 and middle T2 signals. Left cerebellar hemisphere and right caudate nucleus show long T1 and long T2 signals. Right meninges are evenly thickened and significantly strengthened compared with the opposite side. Bilateral mastoid mucous membranes and ethmoid sinus mucous membranes are thickened. An ethmoid sinus cyst is observed in the right posterior group.	There were patches of slightly long T2 signals in the white matter of the bilateral parietal ventricles, and the extracerebral space was widened.	N/A
Broad joints	+	+	N/A	N/A	+
Short stature	+	+	+	+	+
GH deficiency	–	+	N/A	N/A	N/A
Platyspondyly	+	+	+	+	+
Metaphyseal dysplasia	+	+	+	+	+
Bone age	Delayed	Delayed	Delayed	Delayed	Delayed
Skin and mucosa involvement	–	Petechiae/purpura	–	Petechiae	Skin hyperpigmentation, oral ulcers
Infections	–	Suppurative submandibular lymphadenitis and a dental abscess	Sepsis, purulent meningitis, bronchopneumonia, binocular keratitis, binocular uveitis, chronic right suppurative otitis media, urinary tract infection, fungal infection, pericardial effusion, pleural effusion	Pneumonia, respiratory failure	Otitis media
Hematological manifestations	Anemia	Iron deficiency anemia, Immune thrombocytopenia	Anemia, immune thrombocytopenia	Immune thrombocytopenia	Anemia
CBC	Normal	Abnormal	Abnormal	Abnormal	Normal
Hb	Decreased	Decreased	Decreased	Decreased	Decreased
RBCs shape	Normal	Normal	N/A	N/A	Normal
Platelets	Normal	Decreased	Decreased	Decreased	Normal
Immunological manifestations	–	Immune thrombocytopenia present with epistaxis, bleeding from gums, blood in stools	Immune thrombocytopenia present with epistaxis, HLH, hypothyroidism, uveitis	Immune thrombocytopenia present with mucocutaneous hemorrhage	–
Coombs	Negative	Negative	Negative	N/A	N/A
C3\C4	Normal	Normal	Normal	Increased	Decreased
Treatment	Tofacitinib	Tofacitinib	Antibacterial and antifungal treatment, IVIG	Methylprednisolone, Dexamethasone, Azathioprine, IVIG	Steroids, Tofacitinib

N/A, not available.

The diagnosis of SPENCD has long relied on characteristic metaphyseal and vertebral lesions. Skeletal findings often serve as key diagnostic clues. In nearly all assessed patients, skeletal manifestations were evident on radiographs, presenting with either platyspondyly and metaphyseal dysplasia, or only one of these features (5). All our patients demonstrated typical platyspondyly dysplasia. Additionally, P1 exhibited increased intervertebral space; P2 showed markedly uneven spinal density; P3 had delayed ossification center of the thoracolumbar epiphyseal plate center; and P4 presented with uneven vertebral bone density, irregular upper and lower vertebral endplates, and intervertebral space widening (**Figure 1**). P5 presented with platyspondyly and mild metaphyseal dysplasia. Long bone dysplasia was common among the patients. P1 displayed enlargement, invagination, and focal sclerosis in the bilateral proximal humeral metaphyses, femora, fibulae, and tibiae. A similar pattern was observed in bilateral partial rib metaphyses (**Figure 1C**). P2 exhibited bone destruction in the distal radius, ulna, and femora (**Figure 1D**). P3 showed unevenly increased pelvic bone density, rough superior iliac margins, bilateral groin irregularity, and uneven sacral bone density (**Figure 1E**). P4 had roughness along the superior iliac margin, irregular bilateral groin contours, and uneven sacral bone density (**Figure 1F**). Two patients presented with pelvic abnormalities. All patients had delayed bone age, and P2 was also diagnosed with growth hormone deficiency. In addition, P3 exhibited delayed tooth eruption.

### 3.2 Developmental and neurological manifestations

Neurological manifestations are common in ACP5-associated disease. P1 achieved normal motor and mental developmental milestones. However, she developed progressive spasticity and gait abnormalities, including a backward-leaning posture, dragging gait, and slight external rotation of the left foot. She was unable to jump or stand on tiptoes or heels, but retained the ability to squat and to climb stairs independently. Mild to moderate intellectual developmental delay was observed in two patients (P2 and P3). P2 exhibited global developmental delay accompanied by spasticity in all limbs, delayed speech, and delayed independent walking. Prior to six months of tofacitinib treatment, he only spoke monosyllables since age one, with his first words (“Mama” and “Papa”) emerging at 60 months. Spastic paraplegia and upper limb hypertonia impaired hand function. P3 began walking with assistance at age 2 and independently at 2.5 years. Other neurological features, such as ataxia, seizures, or psychosis, were absent in all five patients, and electroencephalograms were normal.

Cranial CT imaging revealed intracranial calcifications in four patients, involving the basal ganglia, pons, cerebellar dentate nuclei, and gray-white matter junction to varying degrees (**Figure 1G**). No cranial CT examination was performed for P5. Intracranial calcifications were observed in patients presenting with neurological manifestations, including spasticity and developmental delay, as well as autoimmune features.

### 3.3 Autoimmunity and immunodeficiency in patients

Immune dysregulation was the most common presenting manifestation in our patients. Immunodeficiency in P1 was mild, and she did not experience recurrent infections. P2 developed suppurative submandibular lymphadenitis and a dental abscess. P3 experienced sepsis, purulent meningitis, bronchopneumonia, binocular keratitis, binocular uveitis, chronic right suppurative otitis media, urinary tract infection, fungal infection, pericardial effusion, and pleural effusion. P4 had recurrent pneumonia, with one episode progressing to respiratory failure. Autoimmune thrombocytopenia (AITP) was the most common autoimmune manifestation, occurring in three patients (P2, P3, and P4). P2 and P4 exhibited spontaneous bleeding and splenomegaly secondary to AITP. P2 presented with petechiae, epistaxis, gingival bleeding, and hematochezia. P3 had recurrent epistaxis, and P4 had recurrent petechiae. Additionally, P3 had hypothyroidism, characterized by decreased free T3 (1.490 pmol/L), decreased free T4 (11.630 pmol/L), and elevated thyroid-stimulating hormone (43.780 mIU/L). Thyroid color Doppler ultrasound revealed mildly increased blood flow signals in the left lobe. P5 presented with generalized rash, swelling of the right knee joint, and oral ulcers, with laboratory investigations revealing strongly positive antinuclear antibodies (ANA) in a homogenous pattern, hypocomplementemia, and anemia, a constellation of findings resembling systemic lupus erythematosus. Both P3 and P5 fulfilled the EULAR/ACR classification criteria for systemic lupus erythematosus (SLE). Serologically, four of the five patients (P1, P2, P4, and P5) showed autoantibody positivity. In addition, P4 was positive for anti-dsDNA and anti-nucleosome antibodies, and P3 was positive for anti–Ro-52 antibody (**Table 1**). AITP often necessitates intensive therapies, including corticosteroids, intravenous immunoglobulin, rituximab, and/or splenectomy (5). P2 received steroid therapy (tapering doses) and tofacitinib for approximately 18 months, achieving a normal platelet count [226×10^9^/L (reference range: 150–400×10^9^/L)] and subsequently discontinuing steroids for several months. P3 was managed effectively with antibacterial and antifungal agents plus IVIG. P4 was treated with methylprednisolone, dexamethasone, and IVIG, after which platelet counts normalized.

In summary, P1 showed mild immunological abnormalities with autoantibody positivity and no recurrent infections; P2 developed suppurative lymphadenitis/dental abscess and AITP requiring immunosuppression and JAK inhibitor therapy; P3 had severe infections and fulfilled criteria for SLE with additional autoimmune manifestations; P4 experienced recurrent pneumonia and AITP requiring corticosteroids/IVIG; and P5 presented with an early-onset SLE-like phenotype treated with corticosteroids and JAK inhibition.

Immunophenotyping in patients with ACP5-associated spondyloenchondrodysplasia has revealed variable abnormalities across T, B, and NK cell compartments, including reduced naïve T cells, altered B-cell maturation, and decreased NK cell numbers (5). Lymphocyte subset analysis in our patients was consistent with previous reports describing heterogeneous abnormalities in these immune cell populations. P1 exhibited low T cells, low NK cells, and elevated B cells. P2 showed increased T cells, normal B cells, and decreased NK cells. P3 had reduced T cells, normal B cells, and low NK cells. P4 demonstrated decreased T and B cells. P5 had normal total counts of T, B, and NK cells (**Table 2**). However, longitudinal immunological follow-up and additional functional readouts were not available for all patients, which limits cross-patient comparisons beyond baseline profiling.

**Table 2:** Comparison of the immune cell subpopulations of SPENCD patients.

Items	Patient 1	Patient 2	Patient 3	Patient 4	Patient 5
Cellular immunity					
CD3 T lymphocytes	Decreased (R)	Increased (A&R)	Decreased (R)	Decreased (R)	Normal
CD4 T helper	Decreased (A)	N/A	N/A	Decreased (R)	N/A
CD8 T cytotoxic	Decreased (A)	N/A	N/A	Decreased (R)	N/A
CD4/CD8 Ratio	N/A	N/A	Decreased	N/A	N/A
CD19 B lymphocytes	Increased (R)	Normal (A&R)	Normal (A)	Decreased (R)	Normal
CD16 NK cells	Decreased (A)	Decreased (A&R)	Decreased (A&R)	N/A	Normal
Humoral Immunity					
IgG	Normal IgG	Elevated IgG	Normal IgG	Elevated IgG	Normal IgG
IgM	Normal IgM	Normal IgM	Normal IgM	Elevated IgM	Normal IgM
IgA	Normal IgA	Elevated IgA	Elevated IgA	Elevated IgA	Normal IgA
IgE	Elevated IgE	N/A	Elevated IgE	Elevated IgE	N/A

A, absolute count; R, relative count.

### 3.4 Identification of four novel *ACP5* mutations

**Figure 2:**
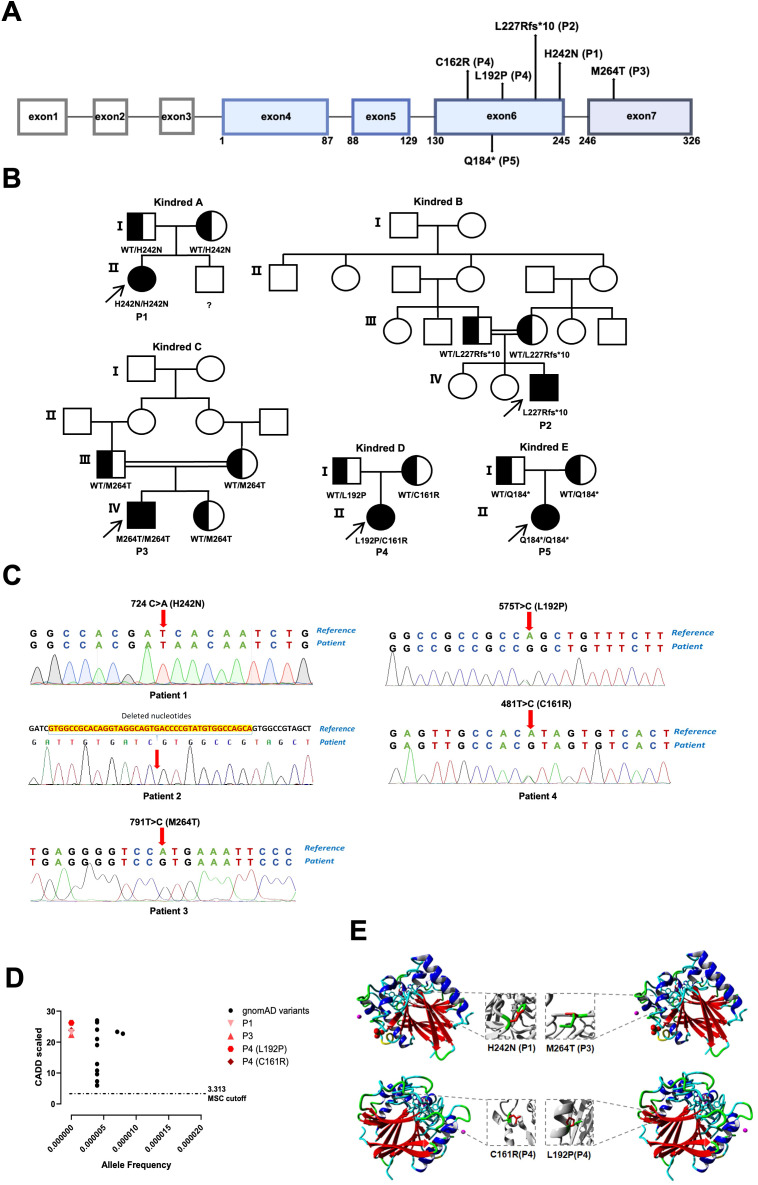
Genetic characteristics of five SPENCD patients. **(A)** Distribution of *ACP5* variants identified in five patients. **(B)** Pedigrees of five unrelated kindreds **(A–E)**; probands are indicated by arrows. **(C)** Representative Sanger sequencing chromatograms confirming the variants. **(D)** CADD scores of the four missense variants plotted against allele frequencies in gnomAD; the dashed line indicates the mutation significance cutoff (MSC). **(E)** Predicted 3D protein structures showing the locations of the four missense variants.

In this study, we identified four homozygous *ACP5* mutations and one compound heterozygous mutations in *ACP5*. Two of the five patients had consanguineous parents (P2 and P3). Among the six *ACP5* mutations identified in five patients, four were novel, while the M246T and Q184* mutations have been reported previously (15). Notably, Q184*, a nonsense mutation introducing a premature stop codon at amino acid 184, was formerly described only as part of a compound heterozygous genotype, whereas our patient is homozygous for this variant. Four missense mutations were identified: p.H242N (c.724C>A) in P1, p.M264T (c.791T>C) in P3, p.C161R (c.481T>C) and p.L192P (c.575T>C) in P4. P2 carried a frameshift deletion (c.680_720del), resulting in a premature stop codon at position 10 downstream (p.Leu227Argfs*10). P4 had a compound heterozygous mutation: p.C161R (c.481T>C) inherited from her mother and p.L192P (c.575T>C) was from her father (**Figures 2B, C**). These six mutations were distributed across exons 6 and 7 (**Figure 2A**). All missense variants were predicted to be deleterious, with CADD scores above the MSC threshold and altered protein structure predictions (**Figures 2D, E**).

### 3.5 Immunologic and clinical response with JAK inhibitor target treatment

**Figure 3:**
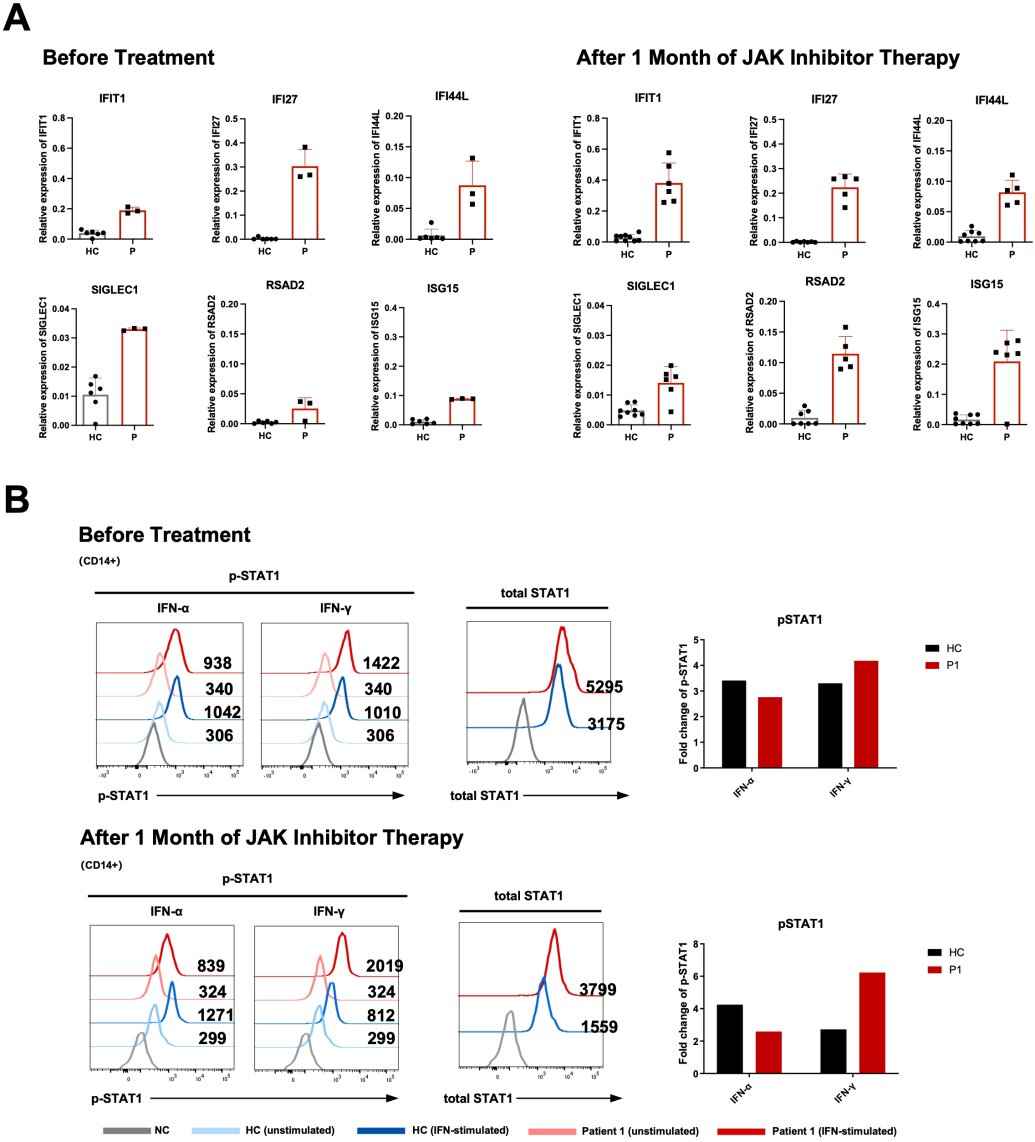
Persistent type I interferon signature and STAT1 hyperactivation in Patient 1 before and after JAK inhibitor treatment. **(A)** ISG expression in whole blood from Patient 1 before treatment and 1 month after JAK inhibitor therapy, compared with healthy controls (HC). Red bars indicate Patient 1, and gray bars indicate HC. HC represent biological replicates (n = 2–3); patient values are descriptive (biological n = 1 per time point). Dots indicate repeated measurements from the same blood draw. **(B)** STAT1 signaling responses in CD14^+^ monocytes from Patient 1 and healthy controls (HC) before treatment and 1 month after therapy. Representative histograms show unstimulated and IFN-stimulated p-STAT1 responses (IFN-α and IFN-γ) and total STAT1 expression. Numbers indicate median fluorescence intensity (MFI). Bar graphs summarize fold changes in p-STAT1 relative to the corresponding unstimulated controls. NC indicates negative control.

Prior studies on SPENCD have concentrated on clinical features with minimal immunological analysis. To delineate the immune profile, we examined immune cell subsets and function in P1 before and after JAK inhibitor treatment. P1 was selected for early autoimmune indications, including positive autoantibodies and elevated serum IFN-α. Interferon-stimulated genes (ISGs) expression (e.g., IFIT1, IFI44L) was higher than in controls, persisting post-treatment (**Figure 3A**). Notably, in P1, elevated ISG expression was also observed at approximately 1 year after initiation of JAK inhibitor therapy (**Supplementary Figure 4**). Enhanced STAT1 phosphorylation after IFN-γ and elevated total STAT1 indicated JAK-STAT pathway activation (**Figure 3B**). Consistently, STAT1 signaling responses in T, B, and NK cells are shown in **Supplementary Figure 2**. Collectively, these findings were consistent with a type I interferon signature in P1.

**Figure 4:**
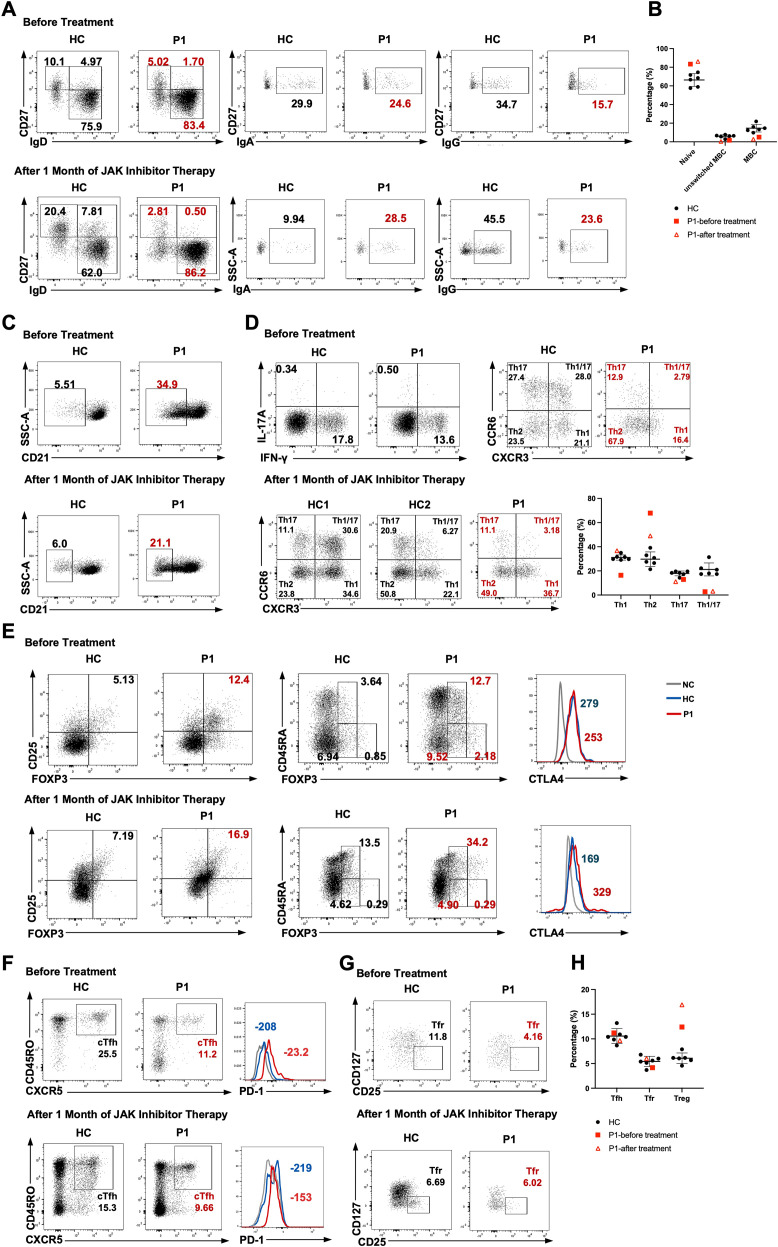
Altered B- and T-cell subset composition in Patient 1 before and after JAK inhibitor treatment. **(A)** Gating overview for B-cell subsets and isotype expression within switched memory B cells. **(B)** Frequencies of naïve, unswitched, and switched memory B cells in Patient 1 compared with pediatric healthy controls before and after treatment (3 years 10 months to 9 years; n = 6, 3 males/3 females). **(C–G)** Flow-cytometric analyses of CD21^low^ B cells and CD4^+^ T-cell subsets (Th1/Th2/Th17, Treg, cTfh, and Tfr) in Patient 1 before treatment and 1 month after therapy. **(H)** Frequencies of cTfh, Tfr, and Treg in Patient 1 compared with pediatric healthy controls before and after treatment (3 years 10 months to 9 years; n = 6, 3 males/3 females). Representative HC plots in panels **(A, C–G)** are from an adult healthy donor analyzed in parallel with P1; pediatric controls are shown as reference data in panels **(B, D, H)**.

We therefore proceeded to analyze lymphocyte subset composition and function. Proportions of T lymphocytes, naïve CD4^+^, CD8^+^, and γδT cells were diminished in P1 (**Table 3**). Total B cell counts remained normal, but transitional B cells increased, and NK cells slightly declined. Altered B cell maturation featured elevated naïve B cells, decreased unswitched/switched memory B cells, and reduced IgG^+^ switched memory B cells (MBC) (**Figures 4A, B**). CD21^low^ B cells were markedly increased, associated with autoimmunity (**Figure 4C**) (16, 17). Immunophenotypic analysis of P1 showed diminished IFN-γ-producing T cells; Th1 and Th17 percentages were lower than healthy controls pre-treatment (**Figure 4D**). Treg proportions were increased both before and after therapy, with levels exceeding those observed in controls at both time points (**Figure 4E**). Circulating T follicular helper (cTfh) cell frequencies were reduced, whereas the proportion of PD-1^+^ cells within the Tfh compartment was increased (**Figure 4F**). Tfr cells were modestly decreased prior to treatment, coinciding with elevated PD-1 expression (**Figure 4G**). Age- and sex-matched pediatric controls were not available for all assays; therefore, adult healthy donors were used for experiments requiring fresh blood, whereas pediatric control data are shown as reference in the summary plots in (**Figures 4B, D, H**) (18).

**Table 3:** Comprehensive analysis of peripheral lymphocyte subsets.

lymphocytes tested	Percentage (%)	Percentage reference range	Absolute numbers (cells/ul)	Absolute numbers reference range
Total T cells	54.5	59.50-75.56	746.7	1480.28-2847.32
CD8+T cells	21.9	19.70-32.04	300	552.62-1127.28
CD8+ Naïve T cells	67.4	38.03-79.08	202.2	293.36-768.42
CD8+ TEMRA T cells	19.5	1.30-22.85	58.5	9.05-209.78
CD8+ CM	9.3	11.91-36.87	27.8	79.59-350.41
CD8+ EM	3.9	1.11-14.51	11.6	7.90-104.18
CD4+T cells	30.1	28.49-41.07	412.9	767.26-1592.48
CD4+ Naive T cells	55.1	40.75-72.70	227.5	338.68-1036.97
CD4+ TEMRA	0.5	0.00-1.47	2.2	0.00-16.71
CD4+ CM	30.5	21.66-52.74	125.9	232.09-600.93
CD4+ EM	13.9	1.90-9.20	57.4	20.54-96.75
TCRαβ+DNT	3.7	0.19-2.43	27.7	3.77-49.48
γδ T cells	8.1	7.00-19.60	60.7	133.70-427.77
Total B cells	29.6	10.46-21.77	404.8	303.52-777.25
Memory B	13.3	8.61-20.19	53.8	37.69-777.25
Naïve B	51.9	52.04-75.78	210.1	171.45-469.28
Transitional B	24.5	3.41-11.17	99.2	14.36-59.62
Plasmablasts B	6.7	0.80-9.75	26.9	3.87-39.83
NK cells	15.9	7.83-20.99	217.7	227.47-667.76
CD4:CD8	1.38	1.02-2.05		

CM, central memory; EM, efector memory.

After one month of tofacitinib treatment, no overt clinical improvement was observed, and serum IFN-α levels as well as ISGs expression remained elevated (**Figure 3A**). STAT1 signaling responses were largely unchanged compared with baseline, characterized by enhanced STAT1 phosphorylation following IFN-γ stimulation, increased total STAT1 expression, and reduced responsiveness to IFN-α (**Figure 3B**). In contrast, the proportions of IFN-γ- and IL-17A-producing T cells returned to levels comparable to those of controls (**Figure 4D**). The frequency of CD21^low^ B cells was significantly reduced compared with baseline but remained higher than in controls (**Figure 4C**). The partial normalization of immune cell composition suggests an improvement in immune dysregulation (**Supplementary Figure 3**).

After four months of JAK inhibitor treatment, P1 self-discontinued the medication for over half a year. Her anemia and positive antinuclear antibody persisted, but lymphopenia resolved. Brain CT revealed that symmetrical calcification in the bilateral lenticular nucleus showed no significant change compared with the scan one year prior, while new scattered calcifications in the bilateral frontal and parietal lobes had increased. P2 and P5 were patients from India who both received JAK inhibitor therapy after diagnosis. P2, diagnosed with immune thrombocytopenia, was treated with tapering doses of steroids and tofacitinib (2.5 mg twice daily), achieving normalization of platelet counts. He has remained steroid-free for several months and achieved four developmental milestones following the initiation of tofacitinib. P5 received steroids combined with tofacitinib (2.5 mg twice daily) and was followed for 11 months, during which she remained clinically stable except for a single self-limiting episode of recurrent arthritis. Both these patients are continuing with tofacitinib therapy.

## 4 Discussion

SPENCD is a form of interferonopathy marked by skeletal dysplasia, neurological involvement, and immune dysfunction. This study describes five patients harboring six mutations in the *ACP5* gene, four of which are novel. Common clinical features included spinal and long bone metaphyseal dysplasia, delayed bone age, and intracranial calcification, aligning with the classic presentation of SPENCD. Patient heterogeneity was evident in the spectrum of immune dysfunction, manifesting as immunodeficiency and autoimmunity. Patient 1 exhibited suspected autoimmune hepatitis and immunodeficiency. Patient 2 developed suppurative submandibular lymphadenitis, dental abscess, and AITP. Patient 3 was diagnosed with SLE, autoimmune hypothyroidism, and AITP. Patient 4 experienced recurrent pneumonia and AITP. P5 was diagnosed with SLE in early childhood. The co-occurrence of immunodeficiency and autoimmunity in these cases underscores the importance of early recognition of autoimmune manifestations across multiple systems in SPENCD patients. Given the rarity of SPENCD and constraints in sample availability, detailed immunophenotyping and functional experiments could be performed mainly in P1. Therefore, mechanistic conclusions should be interpreted cautiously and may not fully capture inter-individual variability.

All reported pathogenic variants in *ACP5* are located between exons 4 and 7. Among the six mutations identified in our five patients, five were situated in exon 6 and one in exon 7 (**Figure 2A**). Notably, individuals carrying the same variant exhibited heterogeneous clinical severity and age of onset, consistent with previous observations that mutation type (missense, nonsense, or deletion) does not predict clinical phenotype or disease severity.

Biallelic mutations in *ACP5* are known to result in reduced expression and activity of tartrate-resistant acid phosphatase (TRAP) in serum and leukocytes, leading to increased phosphorylation of osteopontin (OPN). Dysregulated OPN has emerged as a potential unifying mediator linking skeletal abnormalities and immune dysregulation, as it is expressed in both osteoclasts and immune cells and has been implicated in the pathogenesis of autoimmune diseases (19, 20). In patients with SPENCD, aberrant OPN signaling may skew dendritic cell differentiation toward a proinflammatory phenotype that favors Th1 responses and enhanced inflammatory activity (2). In addition, excessive intracellular phosphorylated OPN has been proposed to amplify Toll-like receptor 9 (TLR9) signaling in plasmacytoid dendritic cells (pDCs), leading to increased production of interferon (IFN)-α and contributing to autoimmune manifestations (21). Consistent with this model, enhanced OPN phosphorylation in antigen-presenting cells has been associated with elevated IFN-α release, a hallmark of type I interferonopathies (2). This mechanistic framework is consistent with the pronounced type I interferon signature observed in our patient cohort. However, we did not directly assess TRAP activity, OPN phosphorylation, or downstream components of the OPN pathway, primarily due to limited sample availability and the retrospective nature of several cases. These analyses will be important in future studies to more directly link *ACP5* genotype to downstream signaling.

Recent studies have highlighted the therapeutic potential of JAK inhibitors in SPENCD, particularly with respect to neurological manifestations and developmental delay, likely through attenuation of interferon signaling (13). Ruxolitinib and baricitinib have also been reported to ameliorate neuroinflammation, autoinflammation, and autoimmune features in patients with type I interferonopathies. In some cases, JAK inhibitor therapy has been associated with catch-up growth, weight gain, and a marked reduction in interferon score (IS), although absolute IS levels often remain elevated. Notably, despite neurological improvement, intracranial calcifications have generally persisted during treatment (22). Treatment-emergent adverse events, most commonly infections and gastrointestinal disorders, have also been reported (23).

In our cohort, JAK inhibition was associated with variable but overall encouraging clinical responses. P2 achieved normalization of platelet counts and attained two additional developmental milestones following initiation of tofacitinib, while remaining steroid-free for several months. P5 remained clinically stable over an 11-month treatment period, with only a single self-limiting flare of arthritis. In P1, lymphopenia resolved after four months of JAK inhibitor therapy; however, treatment was discontinued for more than six months thereafter, and anemia and antinuclear antibody positivity persisted. Follow-up neuroimaging revealed progression of cerebral calcifications, despite stable involvement of the lenticular nuclei.

These findings suggest that JAK inhibition may ameliorate immune dysregulation in patients with biallelic *ACP5* mutations, including cytopenias, lymphocyte abnormalities, and autoimmune manifestations. Importantly, clinical improvement does not necessarily translate into normalization of immunological or radiological endpoints; interferon signatures may remain elevated, and intracranial calcifications may persist during the available follow-up. Consistent with prior reports, neurological improvement can occur even when calcifications do not show radiological reversal. Moreover, catch-up growth in height was not observed in our cohort, supporting the notion that skeletal dysplasia in SPENCD is relatively refractory to interferon blockade and may instead be driven by growth hormone–dependent pathways, particularly in patients with growth hormone deficiency (24).

SPENCD frequently manifests with autoimmune complications, including systemic lupus erythematosus (SLE) and autoimmune thrombocytopenia (AITP), which represent a major component of disease progression. Early detection of CD21^low^ B cells, a population strongly associated with autoimmunity, may therefore facilitate the identification of emerging autoimmune manifestations in patients with *ACP5* deficiency.

In summary, biallelic *ACP5* mutations cause spondyloenchondrodysplasia with immune dysregulation, a rare disorder with substantial clinical heterogeneity. In this small case series, JAK inhibition was associated with improvement of selected immune-mediated manifestations in some patients; however, the evidence remains preliminary given the limited cohort size, heterogeneous concomitant therapies, and short and variable follow-up. Therefore, JAK inhibitors may be considered on an individual basis as a potential therapeutic approach for immune dysregulation, while their long-term efficacy—particularly for neurological and skeletal outcomes—requires confirmation in larger cohorts with standardized longitudinal assessments.

## Data Availability

The original contributions presented in the study are included in the article/**Supplementary Material**. Further inquiries can be directed to the corresponding authors.
