# Supplementary material for: Janus kinase inhibitor therapy for the treatment of spondyloenchondrodysplasia with immune dysregulation due to novel ACP5 variants: a multicenter study
Source: Front Immunol. 2026 Apr 1;17:1788476. doi: 10.3389/fimmu.2026.1788476 (PMC13079001; doi:10.3389/fimmu.2026.1788476)
Supplement: Supplementary file 1 [file DataSheet1.docx]

**
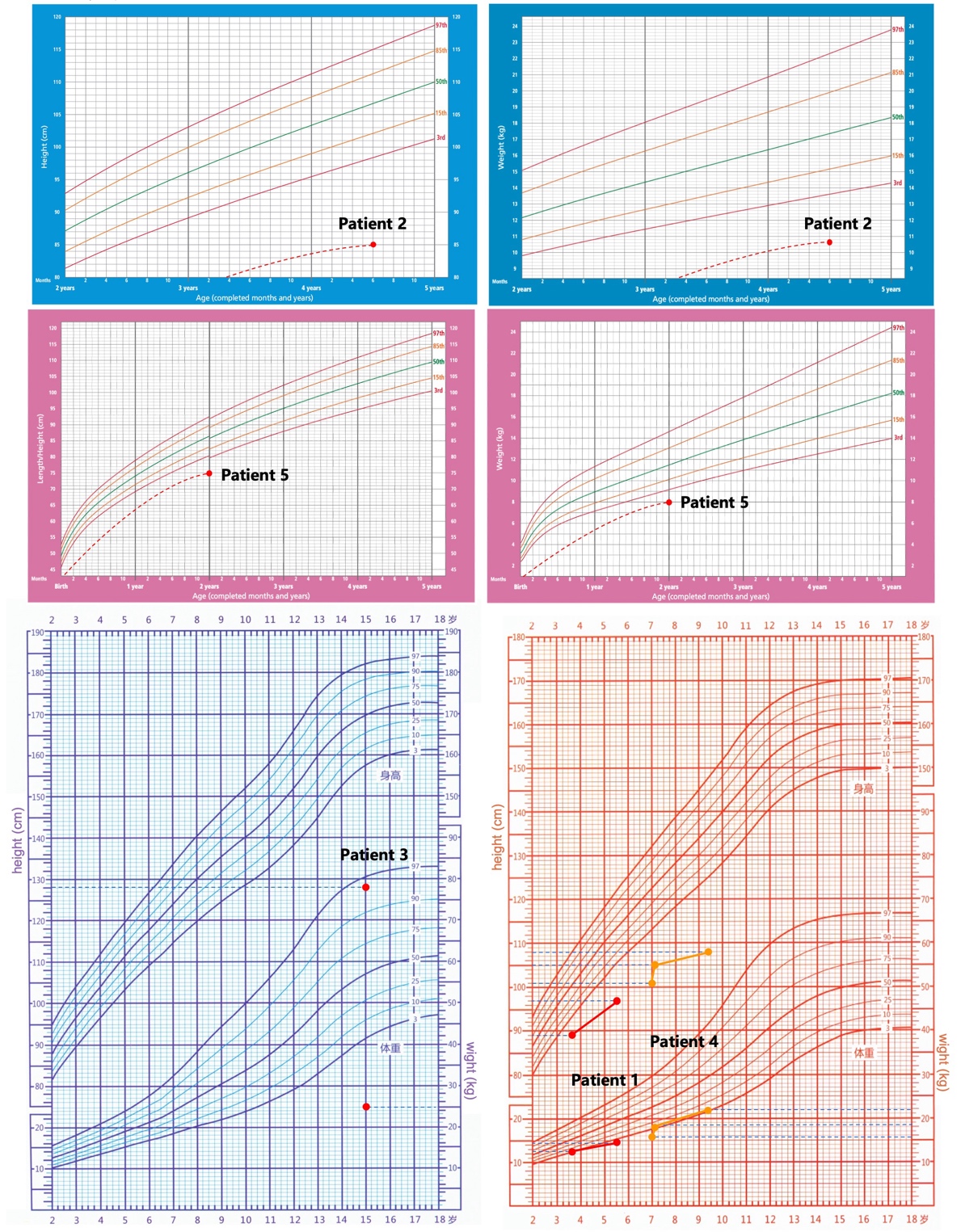
**

**Supplementary Figure 1. Growth trajectories of five patients plotted on age- and sex-specific reference growth charts.**

Longitudinal growth data from individual patients are shown on standardized growth charts using age- and sex-appropriate reference populations. The upper panels display height-for-age and weight-for-age percentiles for boys aged 2-5 years based on World Health Organization (WHO) growth standards, and the middle panels show corresponding percentiles for girls aged 0-5 years. The lower panels present height-for-age and weight-for-age percentiles for boys (left) and girls (right) aged 2–18 years according to Chinese national reference data for healthy children. Dots denote individual measurement time points. For Patient 1 and Patient 4, measurements are additionally connected by solid lines to illustrate longitudinal growth trends.


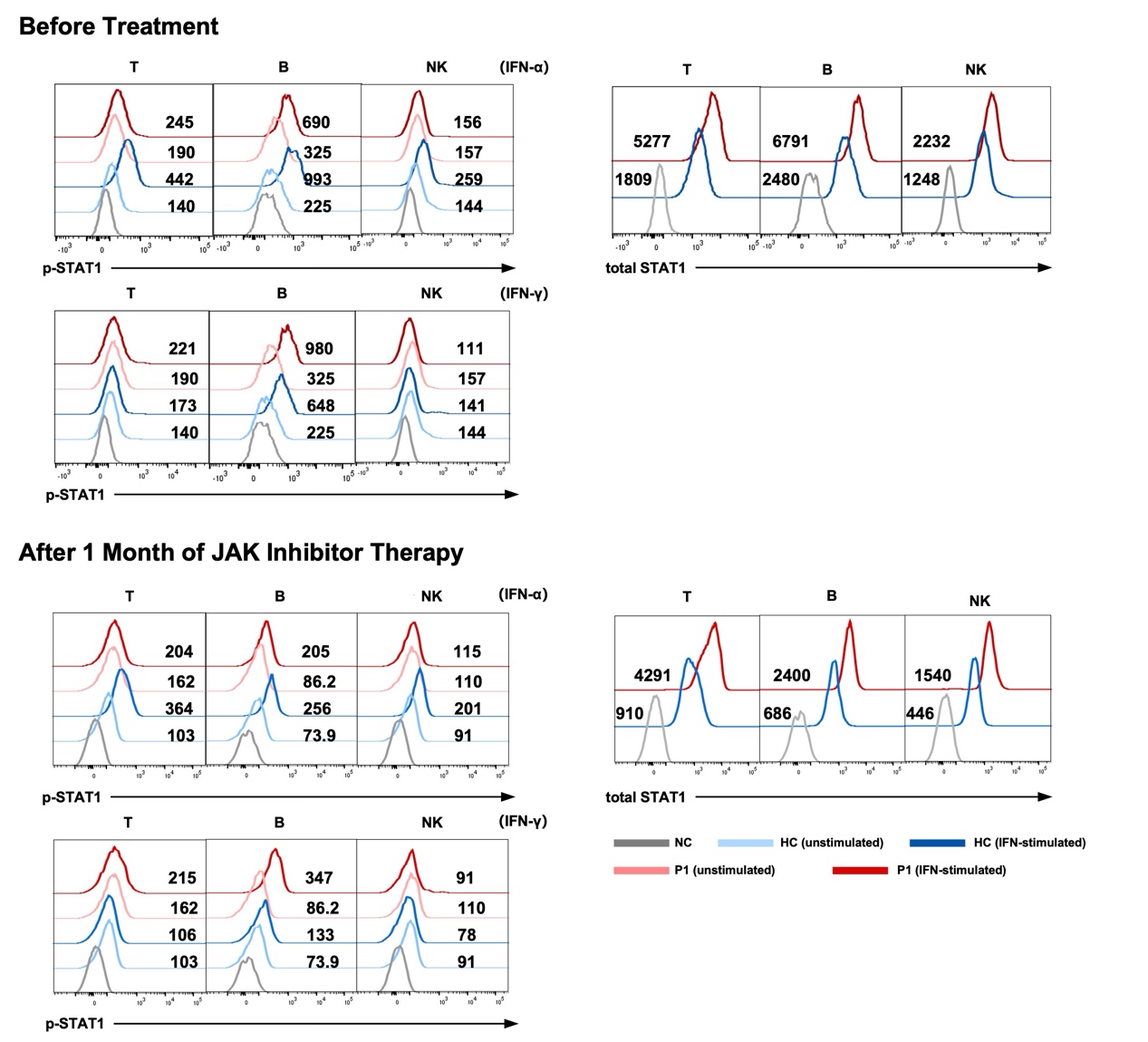


**Supplementary Figure 2. STAT1 signaling responses in T, B, and NK cells from P1 before treatment and after one month of JAKi therapy.** Flow cytometry histograms show phosphorylated STAT1 following stimulation with IFN-α or IFN-γ, as well as total STAT1 expression. Numbers indicate median fluorescence intensity.


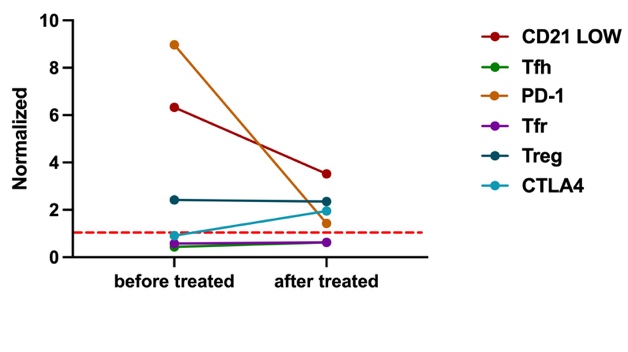


**Supplementary Figure 3. Normalized changes in immune cell subsets in P1 before and after JAKi therapy.** Relative changes in CD21^low^ B cells, Tfh, PD-1 expression, Tfr, Treg, and CTLA-4 expression in P1 before treatment and after one month of JAK inhibitor therapy, normalized to the mean of healthy controls (HC) analyzed in the same experiment. The red dashed line indicates the normalized reference level (value = 1).

**Supplementary Figure 4.** **Persistent type I interferon signature in Patient 1 during JAK inhibitor therapy.** Relative mRNA expression of interferon-stimulated genes (ISGs; IFIT1, IFI27, IFI44L, SIGLEC1, RSAD2, and ISG15) measured by RT–qPCR in whole-blood cells from Patient 1 approximately 1 year after initiation of JAK inhibitor therapy, compared with healthy controls (HC). A treatment interruption of ~6 months occurred during this period. Gene expression was normalized to ACTIN and is presented as relative expression.

**
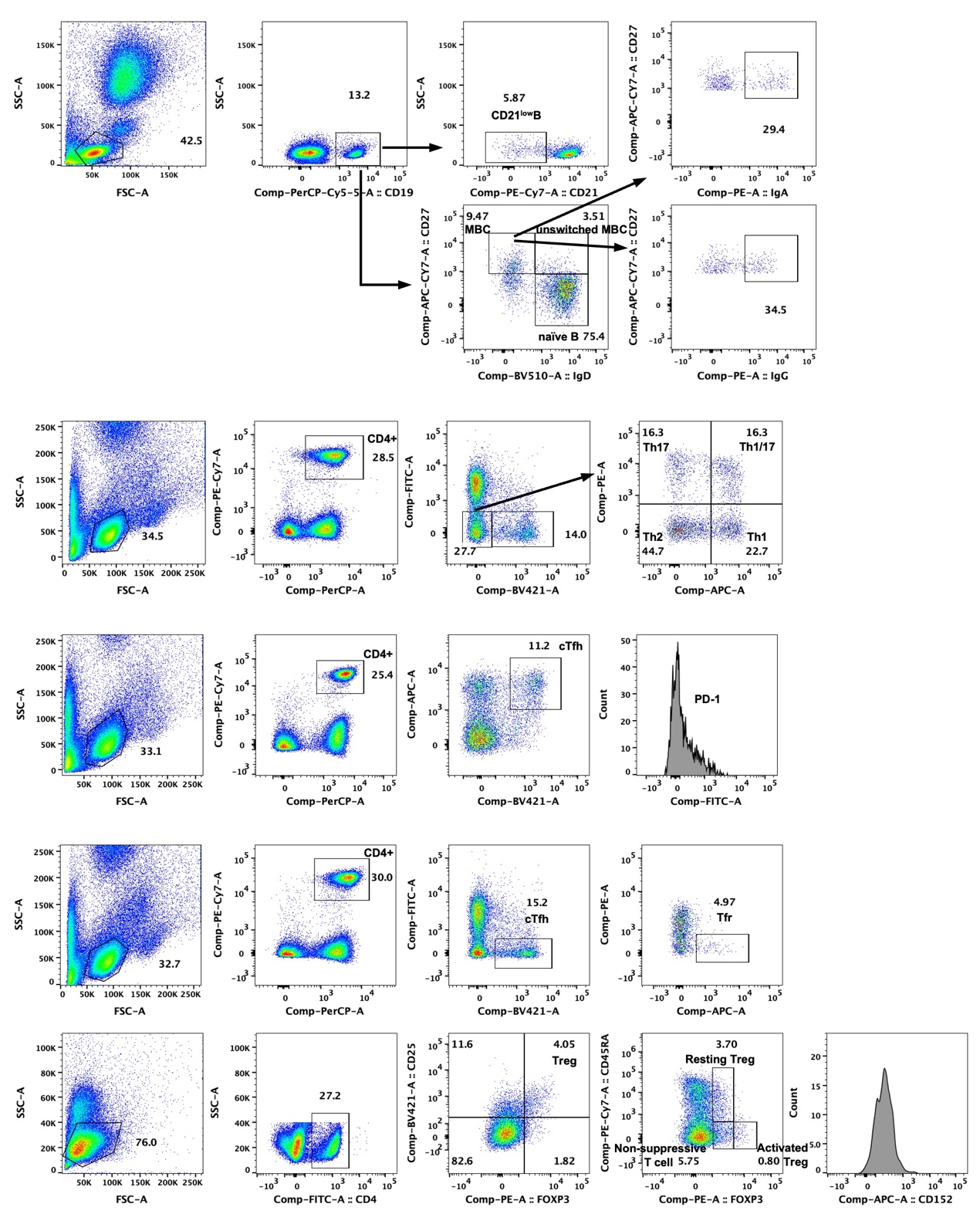
 Supplementary Figure 5.** **Representative flow-cytometry gating strategies.** Representative gating plots are shown for B-cell subsets, Th1/Th2/Th17, cTfh, Tfr, and Treg analyses. Gating was performed as described in the Methods and applied consistently across patients and healthy controls.
